# Notes and News

**Published:** 1894-08-04

**Authors:** 


### Notes and News.

Collected and Communicated.

At the summer meeting of the University Extension Students at Oxford, Dr. J. S. Billings will deliver the inaugural address of the course on hygiene. The chair will be taken by Sir Henry Acland, the Regius Professor of Medicine.

The Leeds charities have recently benefited by a munificent bequest from Mr. John Scott, of Leeds. The sum of £13,000, we are told, is to be divided amongst certain of the benevolent institutions of the town, amongst these being the General Infirmary and the Dispensary.

Dr. S. Weir Mitchell's address to the American Medico-Psychological Association appears in the *New York Journal of Nervous and Mental Disease.* It will prove of special interest to all branches of the medical profession, and has the added interest of appendices in the form of letters from prominent neurologists.

In connection with the British Medical Association at Bristol, Messrs. Armour and Co., the well-known drug manufacturers, desire it to be known that they have prepared a small metal memorandum tablet, suitable to be carried in the waistcoat pocket, which they will present to any medical man attending the congress on application.

Very necessary pressure is being brought to bear, says a contemporary, on the Select Committee which has been considering the Accommodation of the House of Commons, to ensure that the highly important questions of the ventilation and sanitary conditions of the House (which are said to be about as bad as possible), shall be thoroughly gone into.

The Keighley and District Hospital, which has been enlarged, and recently opened by Lady Frederick Cavendish, has been fortunate in securing two generous friends, who have each promised to furnish a ward. The children's ward was selected by one of the gentlemen as the object of his kind intention, but that being already provided for it is proposed that he should endow a cot instead.

The Central Railroad of New Jersey, U.S.A., has recently started a hospital car. It is divided into two compartments, both well fitted for hospital use. Beds for the patients are provided, and medicines and surgical appliances for the sick and injured are in good supply.

Sanitary authorities may possibly be aroused to a better perception of the duties devolving upon them by the results of a case of much importance to all concerned which has just been decided at Tynemouth. Through the non-connection of a house drain with a new sewer an accumulation of sewage arose, resulting in the illness and death of the occupier of the house in question, and cause and effect being absolutely demonstrable, £900 damages have been awarded against the Corporation of Tynemouth as the urban sanitary authority in default.

The outbreak of smallpox in Portland Town, St. John's Wood, has been sudden and severe, but the energetic action taken by Dr. Winter Blyth and the Asylums Board Authorities is having its due effect, and the number of cases is at present abating. So far the disease is not of a specially virulent type. Its curiously rapid spread is easy to understand, as the first case is said to have occurred in a provision shop. The unusual outbreak is putting a great strain upon the vaccinating stations, the applicants numbering as many as 300 a day—a fact which shows that in the face of a recurrence of so loathsome a disease, the anti-vaccinators are not in it.

The General Purposes Committee of the Metropolitan Asylums Board, at the meeting of the managers held last Saturday in the County Hall, Spring Gardens, recommended: "That the committees of management of the various hospitals of the Board be instructed that at the expiration of the existing contracts for window-cleaning, chimney-sweeping, and similar habitual duties, they are to arrange for the discharge of those duties by servants in the permanent employ of the managers, and that for the future no contract for the discharge of such duties by outside labour be entered into." This resolution gave rise to some discussion, not all those present apparently recognising the force of the argument of the committee that allowing a sweep to enter the hospitals and then mix with his fellows outside might spread disease. The proposal was, however, ultimately adopted.

The members of the Public Health Congress have been busy combining work with amusement during the past week. At the invitation of Lord Salisbury, a large party visited Hatfield on Saturday last. On the same day the President and Council were present at a "Creamery Fête" organised in their honour by the Express Dairy Company, at College Farm, Church End, Finchley. Besides the meetings of the different sections, special meetings have been held for discussion on various subjects, among others that of "The Provision of Isolation Hospitals." The final festivity took place at the Guildhall on Tuesday, when the Lord Mayor and Lady Mayoress held a very large reception.

